# The Diet of To-Day

**Published:** 1907-01-26

**Authors:** W. D. Halliburton

**Affiliations:** King's College, London


*To the Editor of* THE HOSPITAL.

### THE DIET OF TO-DAY.

*To the Editor of* THE HOSPITAL.

Sir,—In the excellent summary of my lecture on Diet which appears in your issue of this week, I notice one or two slips your reporter has made which you may think it worth your while to rectify. Thus the percentage of proteid in milk is not ten, but four; cheese is not pure proteid, it is mainly proteid, but contains a certain admixture of fat. Then a few proper names are wrongly spelt; Voight should be Voit, and Loathes should be Leathes. I fear also that the brief *resumé* you published of my remarks on mastication may lead some of your readers to imagine that I advocated "bolting one's food." This would be quite contrary to what I intended to convey, and contrary also to common sense, which I tried to make the keynote of my remarks. What I did condemn was the position in which chewing has been placed by certain ardent advocates who have stated that, with thorough mastication, food can be so increased in value as to render much of the ordinary diet unnecessary. It is as impossible to increase the energy value of any food by dividing it into small pieces as it would be to increase the purchasing value of a sovereign by dividing it into twenty shillings, although small change in both cases may be convenient from other points of view.—Faithfully yours,

King's College, London. W. D. HALLIBURTON.

January 19, 1907.

